# The Activating Transcription Factor 3 (*Atf3*) Homozygous Knockout Mice Exhibit Enhanced Conditioned Fear and Down Regulation of Hippocampal *GELSOLIN*

**DOI:** 10.3389/fnmol.2018.00037

**Published:** 2018-02-20

**Authors:** Chia-Sheng Pai, Pranao K. Sharma, Hsien-Ting Huang, Srivaishnavi Loganathan, Heng Lin, Yu-Luan Hsu, Sarayut Phasuk, Ingrid Y. Liu

**Affiliations:** ^1^Department of Molecular Biology and Human Genetics, Tzu Chi University, Hualien, Taiwan; ^2^Institute of Medical Sciences, Tzu Chi University, Hualien, Taiwan; ^3^Department of Physiology, Taipei Medical University, Taipei, Taiwan; ^4^Department of Physiology, Faculty of Medicine, Siriraj Hospital, Mahidol University, Bangkok, Thailand

**Keywords:** ATF3, fear conditioning, memory, actin, anxiety, posttraumatic stress disorder

## Abstract

The genetic and molecular basis underlying fear memory formation is a key theme in anxiety disorder research. Because activating transcription factor 3 (*ATF3*) is induced under stress conditions and is highly expressed in the hippocampus, we hypothesize that *ATF3* plays a role in fear memory formation. We used fear conditioning and various other paradigms to test *Atf3* knockout mice and study the role of *ATF3* in processing fear memory. The results demonstrated that the lack of *ATF3* specifically enhanced the expression of fear memory, which was indicated by a higher incidence of the freeze response after fear conditioning, whereas the occurrence of spatial memory including Morris Water Maze and radial arm maze remained unchanged. The enhanced freezing behavior and normal spatial memory of the *Atf3* knockout mice resembles the fear response and numbing symptoms often exhibited by patients affected with posttraumatic stress disorder. Additionally, we determined that after fear conditioning, dendritic spine density was increased, and expression of *Gelsolin*, the gene encoding a severing protein for actin polymerization, was down-regulated in the bilateral hippocampi of the *Atf3* knockout mice. Taken together, our results suggest that *ATF3* may suppress fear memory formation in mice directly or indirectly through mechanisms involving modulation of actin polymerization.

## Introduction

Fear is the most profoundly understood emotion in terms of the brain structure and neural circuits involved. Because fear memory expression plays a key role in various anxiety disorders, including posttraumatic stress disorder (PTSD), panic attacks, and phobias, understanding its molecular mechanism is essential (Parsons and Ressler, 2013; Izquierdo et al., 2016). Activating transcription factor 3 (*ATF3*) is induced under stress conditions and is predominantly expressed in the hippocampus. Stress signals induce the *ATF3* through multiple pathways and in a cell type dependent manner. Cumene hydroperoxide activates JNK signal and phosphorylates ATF2 and c-Jun, which subsequently binds to the *ATF3* promoter and activate its expression (Hai and Curran, 1991). Cisplatin, a chemotherapy medicine, induces expression of ATF3 through the p38, ERK and JNK signaling (St. Germain et al., 2010). When human colorectal carcinoma cells are exposed to ultraviolet (UV) or proteasome inhibitor MG132, the *ATF3* is upregulated via p53 signaling (Zhanga et al., 2002). Moreover, the *ATF3* is also induced by extracellular signals including serum, fibroblast growth factor (FGF), epidermal growth factor (EGF) and cytokines (Moore and Goldberg, 2011). In animal models, the *ATF3* is induced in the heart by myocardial ischemia and myocardial ischemia coupled with reperfusion (ischemia-reperfusion) (Brooks et al., 2014). In the liver, the ATF3 can be activated by hepatic ischemia (Rao et al., 2015) and partial hepatectomy (Sandnes et al., 2010). In addition, chemicals including alcohol (Kim et al., 2014), carbon tetrachloride (Chen et al., 1996), and acetaminophen (Wolfgang et al., 1997; Hai et al., 1999) can upregulate expression of the *ATF3*. The *ATF3* can also be activated in the brain by seizure (Pernhorst et al., 2013), in the kidney by renal ischemia-reperfusion (Yoshida et al., 2008), in the skin by wounding (Harper et al., 2005), in the peripheral nerves by axotomy (Tsujino et al., 2000), and in the thymocytes by CD31 (Wu et al., 2014). The *ATF3* is expressed in injured neurons, such as peripheral nerve (Tsujino et al., 2000; Isacsson et al., 2005), optic nerve (Takeda et al., 2000), seizures brain (Chen et al., 1996; Francis et al., 2004), and the CNS glia (Hunt et al., 2004). The ATF3 is upregulated in age-related cognitive decline and neurodegeneration models, that is caused by deficient DNA repair (Borgesius et al., 2011). Previous studies also showed that ATF3 is required for neuron protection (Zhang et al., 2011), neuron regenerating (Campbell et al., 2005) and axon growth (Lindwall et al., 2004). Though the role of ATF3 in stress condition is well studied, its function in fear-induced stress condition and memory formation remains unclear. In this study, we used an *ATF3* knockout mouse (*Atf3^-/-^*) model to investigate the function of *ATF3* in fear memory formation.

Various paradigms have been used to study fear memory formation; the most widely used is classical fear conditioning (Izquierdo et al., 2016). Fear conditioning is a form of associative learning, and the freeze response to conditioning contexts and cues is conserved across species, including humans (Izquierdo et al., 2016). Excess expression of fear memory, indicated by enhanced freezing behavior in mouse models, has been considered a type of PTSD-like symptom. The brain structures involved in fear memory include the cortex, hippocampus, and amygdala (Critchley et al., 2002; Alvarez et al., 2008; Izquierdo et al., 2016). Memory formation involves various cellular and molecular changes including structural alteration of dendritic spines for synaptic plasticity. Dendritic spines, a cellular-level change responsible for synaptic plasticity, are small actin-rich protrusions on the neurites (Niesmann et al., 2011). The organization, dynamics, and density of spines are associated with the strength of synaptic transmission and indicate the efficiency of memory formation (Niesmann et al., 2011; Moczulska et al., 2013).

Changes in the number and morphology of dendritic spines involve molecular-level mechanisms. Studies have described how the involvement of cyclic adenosine monophosphate (AMP) responsive element binding protein (CREB) transcription manipulates the morphology and number of dendritic spines (Sargin et al., 2013; Serita et al., 2017). CREB activation leads to enhanced hippocampal-dependent learning in trace fear conditioning and increased dendritic spine density, indicating the vital role of CREB transcription factors in dendritic spine density (Serita et al., 2017). *ATF3* belongs to a large family of transcription factors including CREB and inducible cAMP early repressor (ICER). The ATF/CREB/ICER transcription factors contains the basic leucine zipper (bZIP) domain that often function as dimers using two extended α–helices to bind DNA and regulate gene expression (Chen et al., 1996). In the hippocampal neurons, the ATF3 has been proved to be a direct target of the CREB. Induction of ATF3 expression by CREB is initiated by calcium entry through synaptic NMDA receptors (Zhang et al., 2011). This stress-induced transcription factor can form homodimers or heterodimers to regulate gene transcription. ATF3 homodimer is a transcription repressor, while forming heterodimer with other protein becomes a transcription activator (Hai and Hartman, 2001). *ATF3* is involved in various physiological and pathological functions, acting as a tumor suppressor or oncogenic gene in various cancers (Yin et al., 2008; Tanaka et al., 2011; Taketani et al., 2012) and regulating glucagon and insulin levels (Lee et al., 2013)^.^ It also regulates arterial dilation during cardiac development (Kehat et al., 2006) and is associated with inflammation (Gilchrist et al., 2008; Tanaka et al., 2011; Hunt et al., 2012; Wang et al., 2012) as well as endoplasmic reticulum (ER)-induced stress responses (Hunt et al., 2012). Furthermore, it has been reported to perform several vital roles in the nervous system, including neuronal growth (Hunt et al., 2012), nerve cell protection (Hunt et al., 2012; Ahlgren et al., 2014) and neurodegeneration (Song et al., 2008).

Under stressed condition, *ATF3* is expressed in the hippocampus (Hunt et al., 2012), which is a vital structure in learning and memory. The expression of *ATF3* in the hippocampus during stress, and its association with various neurological and cognitive functions, led us to hypothesize that *ATF3* is pivotal in regulating the retrieval of stress-induced fear memory. In this study, we used *ATF3* knockout mice (*Atf3^-/-^*) to investigate the function of *ATF3* in retrieval of fear memory.

## Materials and Methods

### Ethics Statement

All protocols used in this study were reviewed and approved by the Institutional Animal Care and Use Committee of Tzu Chi University, Taiwan (No. 103096) and are in compliance with Taiwan Ministry of Science and Technology guidelines on the ethical treatment of animals.

After behavioral testing, the mice were sacrificed immediately after the contextual or tone test was completed (the whole procedure was finished within 3 min). The mice were either perfused for brain sectioning and Golgi staining or decapitated for RNA extraction from the hippocampi. First, the mice were injected with ketamine after the contextual or tone test was finished. The thoracic cavity was opened and transcardially perfused with 120 ml saline. Then the brains of mice were isolated for further sectioning and Golgi staining. For total mRNA extraction, the mice were decapitated by guillotine immediately after the contextual or tone test was finished. And then furry was removed, skulls were opened. The left and right sides of the hippocampus were then separated and bilateral hippocampi were collected respectively for total RNA extraction, because our previous study found that hippocampal gene expression is lateralized (distinct expression patterns for two sides of the hippocampus) (Chung et al., 2015).

### Animals

C57BL/6J wild-type male mice, originally provided by the National Laboratory Animal Center, were purchased and maintained undisturbed in the Lab Animal Center at Tzu Chi University until the behavioral tasks were performed. The *Atf3^+/-^* and *Atf3^-/-^* mice, originally generated by T. Hai (Hartman et al., 2004) and provided by Dr. Hen Lin at Taipei Medical University, Taiwan, were used in the experiment. The *Atf3^-/-^* mice were generated in the 129SVJ background, which contained the clone of *ATF3* gene. And the exon B of the *Atf3* was replaced with Neomycin by direct targeting. Three primers were used in PCR for genotyping and differentiating knockout allele from wild-type allele: 5′-AGAGCTTCAGCAATGGTTTGC-3′ (primer 1), 5′-TGAAGAAGGTAAACACACCGTG-3′ (primer 2), and 5′-ATCAGCAGCCTCTGTTCCAC-3′ (primer 3). And the *Atf3^-/-^* mice were congenic in the background of C57BL/6 for 10 generations (Hartman et al., 2004; Li et al., 2010).

All mice used for the experiments were between 12 and 14 weeks old. The animals were housed in individual plastic and metal cages with *ad libitum* access to food and water under a constant 12-h light/dark cycle. All the experiments on mice and behavioral analyses were double-blinded.

### Locomotor Activity Test

Mice (daytime locomotor activity, WT: *n* = 9; *Atf3^+/-^*: *n* = 10; *Atf3^-/--^*: *n* = 10; nighttime locomotor activity, WT: *n* = 9; *Atf3^+/-^*: *n* = 9; *Atf3^-/-^*: *n* = 9) were placed in an open square chamber (50 cm × 50 cm × 50 cm) for 2 h with no cues or stimuli and were allowed to move freely in the chamber. A video camera and tracking system (TrackMot, Diagnostic & Research Instruments Co., Ltd., Taiwan) were used to measure their moving time and distance.

### Sensory Function Tests

#### Tail Flick Test

Mice (WT: *n* = 7; *Atf3^+/-^*: *n* = 8; *Atf3^-/-^*: *n* = 7) were placed in a 50-mL tube and their tails were exposed to 56°C heat. The time until tail flick was recorded.

#### Pin Prick Test

The experimenter was blinded to the groups of mice. Before testing, the mice (WT: *n* = 6; *Atf3^+/-^*: *n* = 6; *Atf3^-/-^*: *n* = 8) were placed on the testing stage and handed for 15 min. Then the blunted bent gauge needle (at 90° to the syringe) as a stimulus was rubbed to the plantar area of injured hind paw from the heel to the toes in the test. The intensity of stimulus to the plantar was increased by an upward force just sufficient to initiate the withdrawal of paws, but the intensity used was insufficient to penetrate the skin (no scratch or bleeding after pin prick test). The occurrence of paw withdrawal in 10 trials with three applications /trials with an interval of 3–4 s, were summed. The percentage of withdrawal response frequency was calculated by the following formula:

Percentage of response frequency = [Numberofpaw withdrawals/10(numberofapplications)] × 100.

### Fear Conditioning

#### Delay Fear Conditioning

The mice were divided into three groups according to their genotype: WT (*n* = 10), *Atf3^+/-^* (*n* = 10), and *Atf3^-/-^* (*n* = 11). They were placed in the conditioning chamber for 15 min per day for 3 days to allow them to adapt to the novel environment. On Day 4, the mice received three delay fear conditioning (DFC) trials: a 20-s tone (6000 Hz, 85 dB; conditional stimulus [CS]) followed by a 1-s foot shock (2 mA; unconditional stimulus [US]) with an interstimulus interval of 1 min. Twenty-four hours later (Day 5), the mice were placed into the same conditioning chamber for 6 min with no tone or foot shock trial occurring for contextual testing. One hour after the contextual test, the mice were placed in a novel chamber and the tone test was performed as follows: 1 min of neither tone nor shock followed by 6 min of tone (6000 Hz, 85 dB). The fear-conditioning experiments were video recorded, and the freezing behavior (defined as no movement except for breathing) was analyzed using FreezeScan version 1.0 (Clever Sys, Inc., Reston, VA, United States). Moving range in the confined conditioning chamber was defined first and FreezeScan can detect the onset and completion of a freezing behavior of a mouse. Total testing time including freezing time is output as a sequential list, which indicates the occurrences of freezing behavior. The freezing percentage was calculated using the following formula:

%Freezing = (total freezing time/total test time) × 100

The naïve fear-conditioning paradigm was performed as controls. Habituation was performed as described previously. On the training day, neither tone nor foot shock was given. Contextual and tone tests were performed as described previously.

#### Trace Fear Conditioning

The habituation session was performed similarly to the DFC. The mice were divided into three groups (WT: *n* = 15; *Atf3^+/-^*: *n* = 10; *Atf3^-/-^*: *n* = 16) and placed in the conditioning chamber for 15 min per day for 3 days. On Day 4, they underwent three trace fear conditioning (TFC) trials: a 20-s tone (6000 Hz, 85 dB; CS), followed by a 10-s time interval, and then a 1-s foot shock (2 mA; US). Twenty-four hours later, the mice were placed in the same conditioning chamber for 6 min with neither tone nor foot shock for contextual testing. One hour after the contextual test, the mice were placed in a novel chamber for a tone test, which was performed as follows: 1 min with neither tone nor foot shock followed by a 6-min tone (6000 Hz, 85 dB). Freezing behaviors were recorded and analyzed using the FreezeScan software, and the freezing percentage was calculated using the aforementioned formula.

### Morris Water Maze Test (MWM)

A circular pool (diameter: 109 cm, platform height: 21 cm) was filled with water at room temperature (21°C ± 1°C). The water was made opaque using a non-toxic white paint (catalog No. 187203, Palmer Paint Products, MI, United States). Four points equally dividing the pool into four quadrants were chosen, and a round platform (diameter: 10 cm) was placed in the second quadrant. A visible platform test was performed for the first 2 days (8 trials per day). One centimeter of the platform was above water level, and the mice (WT: *n* = 12; *Atf3^-/-^*: *n* = 11) were trained to locate the platform within 60 s on the basis of different cues. The starting point for each trial was randomly selected from among the four quadrants. In the hidden platform test, the platform was placed 1 cm below water level. Every day for 4 consecutive days, each mouse underwent eight trials of 1 min each to locate the hidden platform. If they did not locate the platform, they were guided to it and left there for 10 s so that they could learn its location. A video camera and tracking system (TrackMot) were used to measure the escape latency. On Day 7, a probe test was performed. The platform was removed from the pool and each mouse was allowed to swim freely for 60 s. The percentage of time spent in each quadrant was calculated.

### Radial Arm Maze Test

An octagonal maze was used (diameter: 20 cm; arm dimensions: 35 × 5 × 10 cm), with 0.2 g of peanut butter (Skippy Peanut Butter, Austin, MN, United States) placed at the end of each arm. Three days of habituation sessions were performed. Mice (WT: *n* = 6; *Atf3^+/-^*: *n* = 10; *Atf3^-/-^*: *n* = 7) were allowed to explore freely for 15 min. From days 4 to 8, training sessions were conducted where peanut butter was placed only on predetermined ‘correct arms’ and mice were allowed to explore. Regular chow was restricted 12 h before testing. On the testing day, no peanut butter was placed on the arms. A video camera and tracking system (TrackMot) was used to measure the time the mice spent on the predetermined correct arms.

### Dendritic Spine Staining through Golgi–Cox Staining and Density Measurement

Gogli–Cox solution was prepared in accordance with the Golgi–Cox Staining Protocol for Neurons and Processes^1^. Whole mouse brains were incubated in Golgi–Cox solution for 14 days and the solution was changed every 2 days. After 14 days, the brains were transferred to 30% sucrose and incubated until they sank. Using a vibratome, 60-μm-thick sections of brain were sliced and placed on slides. The washing procedure of Gibb and Kolb (1998) was followed. The slides were then covered using a coverslip with permount and left to dry for 24 h. Samples were observed under an optical microscope and the dendritic spine density was calculated using ImageJ. We measured the density of basal dendrites of pyramidal cells in the CA1 region, since polarized growth of apical dendrites is regulated by cell intrinsic programs, while outgrowth of basal dendrites requires extracellular cue(s) sent from the dentate gyrus. For each brain, 20 sections were analyzed, and for each section, 10 neurons (five from the left hippocampus and five from the right hippocampus) were used to calculate the dendritic spine density. “Measure” function of the ImageJ was used to target the longest dendrite under each field with calibrated scale bar, then the number of spines was counted with the function of “Cell count.” The dendritic spine density was calculated using the following formula:

Dendritic spine density = Total number of spines/length of dendrite.

### Drug Infusion by Stereotactic Injection

At 14 weeks of age, mice (*Atf3*^+/+^: *n* = 5, *Atf3^-/-^*: *n* = 9) were anesthetized using a mixture of ketamine, xylazine, and saline (0.55 mL/25 g) in accordance with the regulations of the Lab Animal Center, Tzu Chi University. Mice heads were restrained in stereotaxic apparatus to ensure movement restriction during surgery. The cannulas were inserted into the third ventricle, just above the hippocampus [anterior-posterior (AP): -2.18 mm from the bregma, dorsoventral (DV): 1.8 mm] and were sealed using acrylic powder. After surgery, the mice were placed in their respective cages to recover for 4 days and were then tested for TFC. Habituation was performed as described in the trace fear-conditioning protocol. Using a Hamilton syringe, the drug cytochalasin D (Sigma–Aldrich, St. Louis, MO, United States, catalog No. C8273), dissolved in dimethyl sulfoxide at a concentration of 25 μg/μL, was infused at a rate of 0.5 μL/min for 1 min immediately after fear conditioning on Day 4. Twenty-four hours later, contextual and tone testing was performed as previously described. A few mice were unable to perform the tasks because of loss of mobility after surgery; the data for these mice are not included. Mice were transcardially perfused, and their brains were incubated in Golgi–Cox staining solution.

### Real-Time Quantitative PCR for Analysis of Gene Expression Levels

After fear conditioning, mice were sacrificed and total RNA was extracted from the hippocampus by using the TRIzol method. Two micrograms of RNA were reverse transcribed using a high-capacity complementary DNA (cDNA) reverse transcription kit (Applied Biosystems, Foster City, CA, United States). Amplification reactions were performed using the PowerSYBR Green Master Mix (Applied Biosystems) in a Roche 480 Real-Time PCR system (Applied Biosystems StepOne, Applied Biosystems). The relative quantitative threshold cycle (ΔΔCt) method was used to analyze the gene expression level. The expression levels of all genes were normalized to the glyceraldehyde 3-phosphate dehydrogenase expression level.

### Statistical Analysis

The data presented in this study were calculated and plotted using the mean as a central tendency with standard error. Two-way analysis of variation followed by Holm-Sidak test was used for all Pairwise Multiple Comparison Procedures (behavioral results for DFC and TFC). One-way analysis of variation followed by Tukey’s test was applied to results of radial arm maze (RAM) and contextual/tone tests after cytochalasin injection. Student’s *t-*test was used to compare results between two groups (results of dendritic spine density). The statistical tests and results were conducted at the 95% confidence level (*p* < 0.05) and 99% confidence level (*p* < 0.01), to ensure the changes were significant. SPSS (IBM, New York, NY, United States) and GraphPad Prism (GraphPad Software, Inc., La Jolla, CA, United States) were used for statistical analysis and to plot the charts, respectively.

## Results

### *Atf3^-/-^* Mice Were Grossly Normal in Morphology, Body Weight, Locomotor, and Nociception (Tail Flick and Paw Test) Functions

**FIGURE 1:**
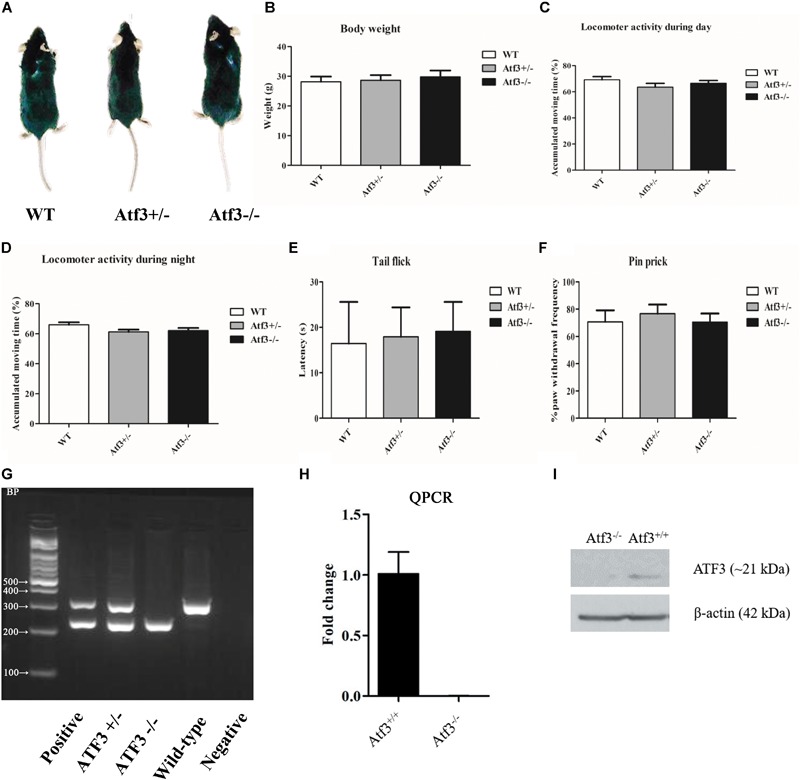
Characterization of the *Atf3^-/-^* mice. No significant difference (*p* > 0.05) was recorded between *Atf3^-/-^* mice and their wild-type littermates in morphology **(A)**, body weight (WT: *n* = 7; *Atf3^+/-^*: *n* = 8; *Atf3^-/-^*: *n* = 9) **(B)**, locomotor activity during the daytime (WT: *n* = 9; *Atf3^+/-^*: *n* = 10; *Atf3^-/-^*: *n* = 10) **(C)** and nighttime (WT: *n* = 9; *Atf3^+/-^*: *n* = 9; *Atf3^-/-^*: *n* = 9) **(D)**, t`1q1ail flick test (WT: *n* = 7; *ATF3^+/-^*: *n* = 8; *Atf3^-/-^*: *n* = 7) **(E)**, pin prick test (WT: *n* = 6; *Atf3^+/-^*: *n* = 6; *Atf3^-/-^*: *n* = 8) **(F)**. Genotyping with PCR was performed for each mouse used for behavioral experiments **(G)**. Neither *ATF3* transcript **(H)** nor ATF3 protein **(I)** was detected in the hippocampus.

To characterize the *Atf3^-/-^* mice and exclude significant deficits in development and sensory functions that might have affected their behavioral performance, we investigated their morphology (**Figure 1A**), body weight (**Figure 1B**), locomotor activity during the daytime (**Figure 1C**) and night time (**Figure 1D**), and nociception functions (**Figures 1E,F**). No significant differences were recorded among the *Atf3* heterozygous, homozygous knockout, and wild-type littermates. DNA extracted from the toes of individual mice was used for PCR genotyping before the breeding and behavioral experiments (**Figure 1G**). Neither *ATF3* transcript (**Figure 1H**) nor ATF3 protein (**Figure 1I**) was detected in the hippocampus.

### The *Atf3^-/-^* Mice Exhibited Enhanced Freeze Responses to Both Delayed Fear Conditioning (DFC) and Trace Fear Conditioning (TFC)

**FIGURE 2:**
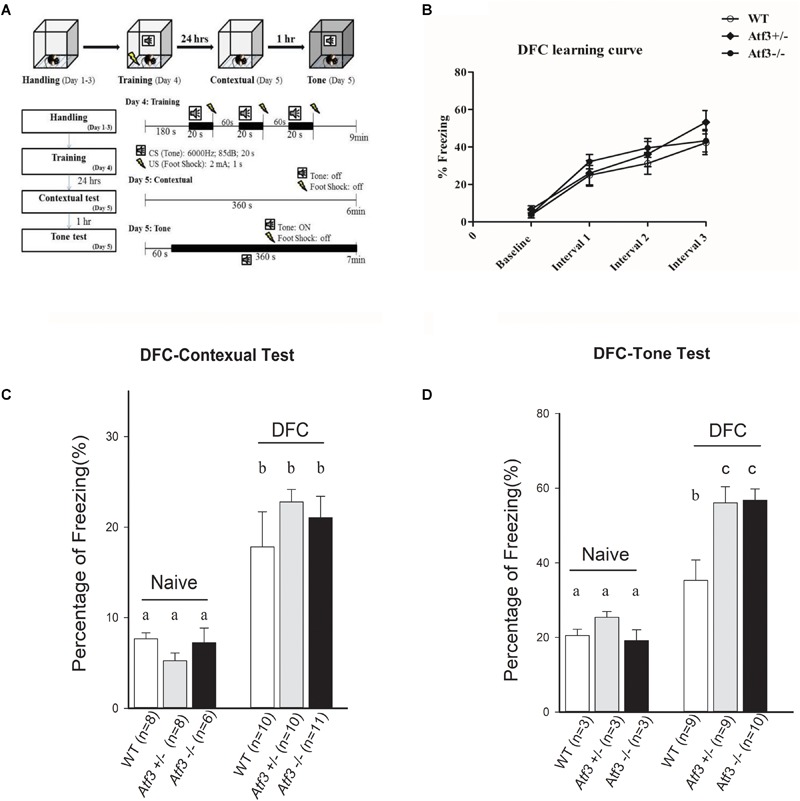
Delay fear conditioning. Mice were trained with delay fear conditioning paradigm and tested their freezing responses to context and tone **(A)**. Compared with their wild-type and heterozygous littermates, the *Atf3^-/-^* mice acquired delay fear conditioning as well **(B)** and retrieved contextual delay fear memory normally (WT: *n* = 10; *Atf3^+/-^*: *n* = 10; *Atf3^-/-^*: *n* = 11) **(C)**. However, the *Atf3^-/-^* mice responded to the tone cue with a significantly higher incidence of freezing behavior (WT: *n* = 9; *Atf3^+/-^*: *n* = 9 *Atf3^-/-^*: *n* = 10) **(D)**. a, b, and c indicate significant difference among groups with different letters (*P* < 0.05).

**FIGURE 3:**
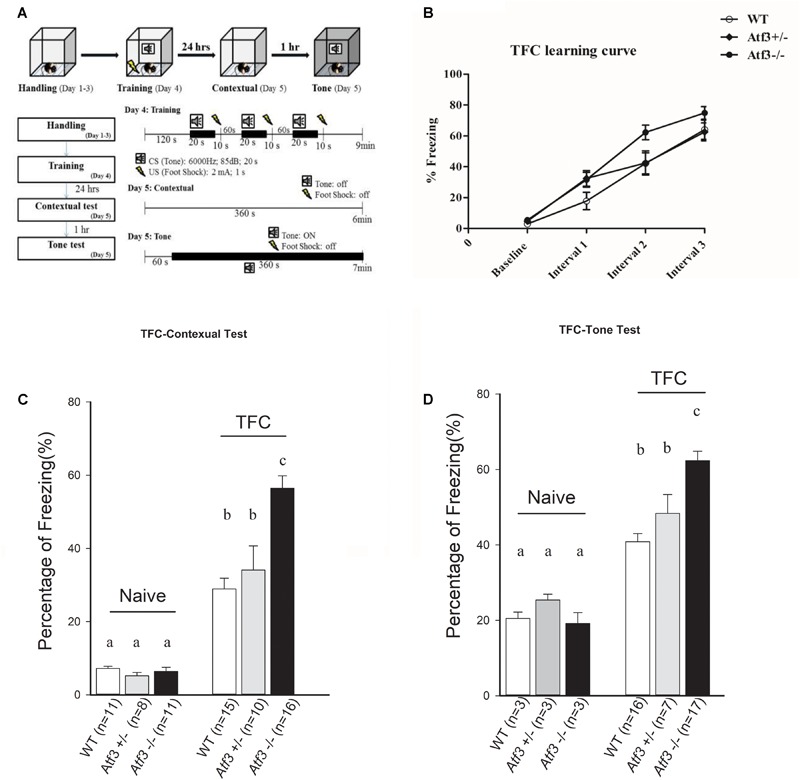
Trace fear conditioning. Mice were trained with trace fear conditioning and tested with their freezing responses to context and tone **(A)**. The *Atf3^-/-^* mice acquired trace fear conditioning as well as their wild-type and heterozygous littermates **(B)**, and responded to both contextual **(C)** (WT: *n* = 15; *Atf3^+/-^*: *n* = 10; *Atf3^-/-^*: *n* = 16) and tone **(D)** (WT: *n* = 16; *Atf3^+/-^*: *n* = 7; *Atf3^-/-^*: *n* = 17) cues with a significantly higher incidence of freezing behavior (*p* < 0.05). a, b, and c indicate significant difference among groups with different letters (*P* < 0.05).

To investigate the role of the *ATF3* gene in fear memory formation, we performed two fear-conditioning tests. Mice were trained with DFC first, in which a tone was followed by an electric foot shock; freezing behavior to context and to tone was measured on the following day (**Figure 2A**). The *Atf3^-/-^* mice underwent DFC (**Figure 2B**) and responded to context (**Figure 2C**), as did their wild-type littermates. Notably, however, the *Atf3^-/-^* mice responded to a significantly greater extent to tone (**Figure 2D**) than their wild-type littermates. We subsequently used another batch of mice and performed TFC, in which mice were fear conditioned using three tone–shock pairs separated by a short temporal interval. TFC is a more complicated training paradigm than DFC; mice must learn not only the association between the tone and the shock but also the temporal interval (**Figure 3A**). The results indicated that the *Atf3^-/-^* mice acquired TFC as thoroughly as their wild-type littermates did (**Figure 3B**), but their freeze responses to both context (**Figure 3C**) and tone (**Figure 3D**) were significantly stronger than they were among the wild-type mice.

### *Atf3^-/-^* Mice Were Normal in Acquisition and Retrieval of Spatial Memory

**FIGURE 4:**
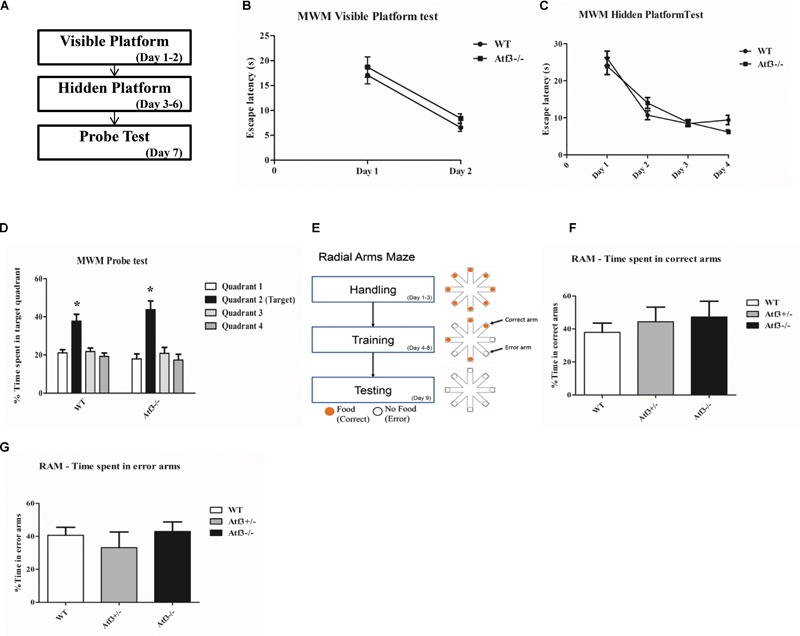
Hippocampal-dependent Morris Water maze and RAM tests. The *Atf3^-/-^* mice performed normally in the Morris water maze tests **(A)**, including in visible platform (WT: *n* = 12; *Atf3^-/-^*: *n* = 11) **(B)**, hidden platform **(C)**, and probe trial **(D)** tests. They also performed normally in the radial arm maze (RAM) test (WT: *n* = 6; *Atf3^+/-^*: *n* = 10; *Atf3^-/-^*: *n* = 7) **(E)**. No significant difference was recorded for time spent in either the correct arms **(F)** or the error arms **(G)**.

Because *ATF3* is a stress-induced transcription factor and fear conditioning is a stressful training paradigm, we subsequently explored whether the stronger memory retrieval response (freezing) of the *Atf3^-/-^* mice to DFC and TFC is specific to fear memory. We trained the *Atf3^-/-^* mice to adapt to the hippocampal-dependent Morris water maze (**Figures 4A–D**) and radial arm maze (RAM) (**Figures 4E–G**). Notably, compared with their wild-type littermates, the *Atf3^-/-^* mice performed normally for the visible platform (**Figure 4B**), hidden platform (**Figure 4C**), and probe trial (**Figure 4D**) tests. No significant differences were recorded among the groups, including for the time they remained on the correct arms (**Figure 4F**) and the time they remained on the error arms (**Figure 4G**) in the RAM test. The results of behavioral phenotyping indicated that the *Atf3^-/-^* mice specifically exhibited a stronger response to the retrieval of fear memory, whereas their performance in spatial tests was normal.

### Density of Dendritic Spine in the Hippocampal CA1 Region of *ATF3^-/-^* Mice Was Significantly Higher Than That of Wild-Type Mice after Retrieval of Contextual Trace Fear Memory

**FIGURE 5:**
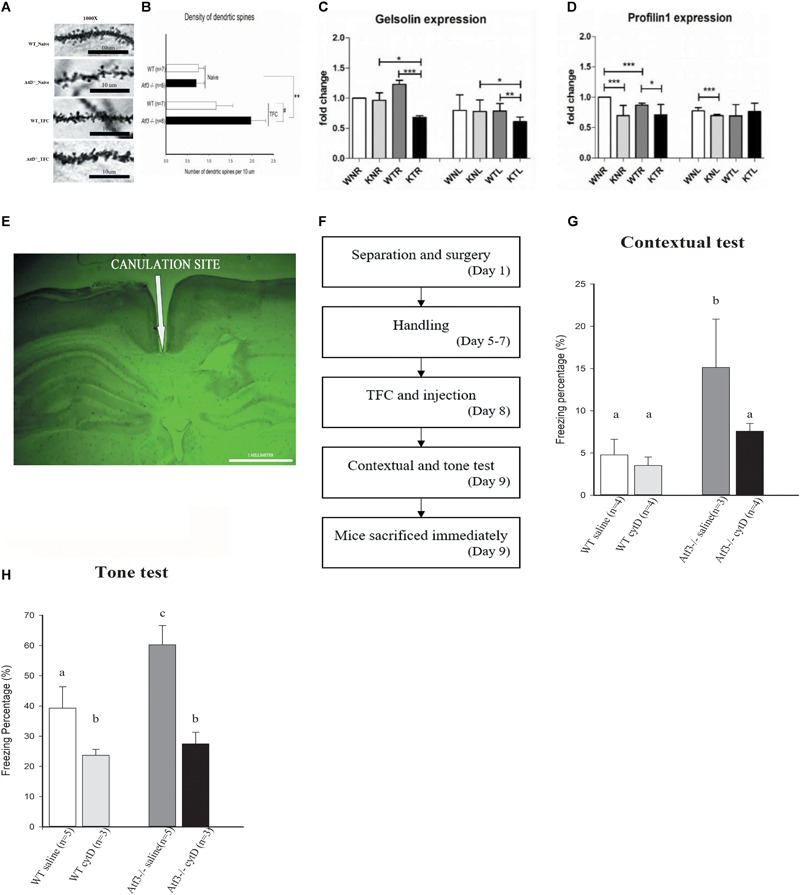
Loss of *ATF3* increases dendritic spine density in the hippocampal region after retrieval of contextual trace fear memory. Inhibition of actin polymerization through intracranial infusion of cytochalasin D reversed the phenotype of *Atf3^-/-^* mice. Microscopic graphs of dendritic spines in the hippocampal CA1 region **(A)**. Quantification of spine density of basal dendrites measured after retrieval of contextual memory for the *Atf3^-/-^* mice and their wild-type littermates with or without trace fear conditioning **(B)**. mRNA expression levels of *Gelsolin* **(C)** and *Profilin 1* **(D)** in wild-type and *Atf3^-/-^* mice before and after training (*n* = 3 for each sample; W, wild type; K, knockout; N, naïve; T, trained groups; L, left hippocampus; R, right hippocampus). Position of cannula placement (AP: –2.18 mm from the bregma, DV: 1.8 mm. **(E)**. Timeline for intracranial infusion and fear conditioning **(F)**. Freezing percentage to context (*n* = 4: WT saline, 4: WT treated with drug, 3: KO saline and 4: KO treated with drug) **(G)** and to tone (*n* = 5: WT saline, 3: WT treated with drug, 5: KO saline and 3: KO treated with drug) **(H)** after injection of cytochalasin D (cytD). The results shown in the figure are plotted as mean ± SE and statistically tested with one-way ANOVA followed by Tukey test and set a confidence level of 99% (*p* < 0.001). a, b, and c indicate significant difference among groups with different letters (*P* < 0.05).

Because the *Atf3^-/-^* mice expressed a PTSD-like, excessive fear response and improved fear memory formation is associated with increase of dendritic spine density (Restivo et al., 2009; Snigdha et al., 2016), we subsequently investigated whether this behavioral phenotype was correlated with changes in hippocampal spine density. We used the Golgi–Cox staining technique to stain brain sections and measure the density of dendritic spines in the CA1 area of the dorsal hippocampi. We measured the density of basal dendrites of pyramidal cells in the CA1 region, since polarized growth of apical dendrites is regulated by cell intrinsic programs, while outgrowth of basal dendrites requires extracellular signaling from the dentate gyrus. In correlation with their behavioral performance, the *Atf3^-/-^* TFC group had a higher dendritic spine density than did the wild-type TFC and naïve groups (**Figures 5A,B**).

### Gelsolin Is Down-regulated in *Atf3^-/-^* Mice and Enhanced Freezing Behavior Is Reversed through Actin Polymerization Inhibition

Because the *Atf3^-/-^* mice exhibited significantly enhanced dendritic spine density in the hippocampal neurons and actin polymerization is the major process involved in dendritic spine formation, we investigated whether *ATF3* directly regulates the actin polymerization process by measuring the messenger RNA (mRNA) levels of two major players, *Gelsolin* and *Profilin 1*. Quantitative reverse transcription polymerase chain reaction (RT-PCR) results indicated that the expression of *Gelsolin*, the gene encoding a severing protein for actin polymerization, was down-regulated in the bilateral hippocampi of *Atf3*^-/-^ mice after fear conditioning (**Figure 5C**). Since more efficient actin polymerization indicates higher freezing response, this indicated that the normal function of *ATF3* is to maintain optimal *Gelsolin* levels to stop actin polymerization at certain point and prevent the formation of excess fear response. By contrast, *Profilin 1* was significantly down-regulated in the bilateral hippocampi of the naïve *Atf3*^-/-^ mice and in the right hippocampus of the TFC-trained *Atf3*^-/-^ mice (**Figure 5D**). Since activation of ATF3 is activity dependent, the expression level changes of Profilin 1 occurred mainly in the naïve, not trained, condition, and was not associated with increase of fear responses indicating that *Profilin1* expression is not regulated by the ATF3. Further investigation is required to understand how *Gelsolin, Profilin 1*, and other genes related to actin polymerization are regulated during the formation of fear memory. Subsequently, we used cytochalasin D, which inhibits the actin polymerization process in a similar fashion to *Gelsolin*, to confirm a correlation between actin polymerization, dendritic spine density, and fear memory expression. The mice underwent surgery 1 week prior to TFC, and stainless steel cannulas were placed at AP (-2.18 mm from the bregma, DV: 1.8 mm) (**Figure 5E**). Mice were allowed to recover for 4 days, and then the TFC paradigm was applied (**Figure 5F**). We determined that the freezing percentages in *Atf3^-/-^* mice injected with cytochalasin D were significantly lower compared with the *Atf3^-/-^* mice treated with saline and were reduced to a percentage similar to that of wild-type sham control. Corresponding results were determined for both the contextual (**Figure 5G**) and tone tests (**Figure 5H**).

## Discussion

Our results demonstrate that the lack of *ATF3* in mice leads to increased freezing behavior in the fear-conditioning paradigm. Additionally, normal responses of the *Atf3^-/-^* mice to other hippocampus-dependent learning paradigms indicate that *ATF3* specifically suppresses stress-induced fear memory. Along with the enhanced freezing behavior results, basal dendritic spine density in the dorsal hippocampal CA1 area of the *Atf3^-/-^* mice was also increased compared with their wild-type littermates. The molecular basis of this phenomenon is reflected by decreased expression of *Gelsolin*, a capping molecule that prevents actin polymerization and dendritic spine protrusion modulation. Hippocampal injection of cytochalasin D, an actin polymerization inhibitor, reduced the freezing behavior of the *Atf3^-/-^* mice to a wild-type mouse level. This suggests that normal *ATF3* may suppress expression of fear memory by directly or indirectly regulating actin polymerization.

Expression of fear memory (fear response) is highly conserved across species, which indicates its importance and survival throughout evolution. Moderate fear memory helps organisms avoid danger and is advantageous for survival, whereas excess fear memory leads to symptoms of anxiety disorders and affects daily life (Izquierdo et al., 2016). The present study suggests that *ATF3* is a possible ‘brake’ to prevent overexpression of fear memory. Lacking *ATF3* gene may lead to enhanced fear response observed in patients affected with anxiety disorders including PTSD.

Previous studies have reported the importance of dendritic spines under stress conditions, associative learning, and memory formation (Niesmann et al., 2011; Leuner and Shors, 2013; Moczulska et al., 2013; Sargin et al., 2013) and have reported that dendritic spine density increases after different fear-conditioning paradigms (Giachero et al., 2013; Heinrichs et al., 2013; Maroun et al., 2013; Keifer et al., 2015). Additionally, studies have determined the importance of CREB transcription factors in regulating dendritic morphology during learning (Middei et al., 2012; Sargin et al., 2013; Serita et al., 2017). A recent study reported that constitutive CREB activation is involved in both short-term and long-term memory formation (Serita et al., 2017). Another study determined that inactivation of CREB not only results in dendritic spine collapse but also affects downstream molecules such as α-amino-3-hydroxy-5-methyl-4-isoxazolepropionic acid receptor translocation and actin-binding proteins (Middei et al., 2012). Apart from its role in neuronal plasticity, CREB also plays a role in activating other genes involved in neurogenesis and neuroprotection. A study reported that because *ATF3* is a direct target of CREB, it is activated by CREB to protect neurons from death induced by the stimulation of extrasynaptic *N*-methyl-D-aspartate receptors (Zhang et al., 2011). Another study suggested that *ATF3* acts as a transcriptional repressor and is involved in microtubule stabilization (Ahlgren et al., 2014). These previous studies support our finding that actin polymerization and dendritic spine changes in the *Atf3* knockouts are important in moderating the expression of fear memory.

Actin polymerization and depolymerization are vital to dendritic spine morphogenesis and dynamics (Matus, 2000; Hotulainen et al., 2009, Mantzur et al., 2009). *Gelsolin* is an actin-modulating/severing protein that promotes nucleation in actin polymerization by binding to the barbed end of actin filaments and preventing its progression (Khaitlina et al., 2013); this means that the protein is crucial to dendritic spine remodeling in synaptic plasticity (Hlushchenko et al., 2016). Moreover, *ATF3* has been determined to bind to the regulatory regions of Gelsolin, resulting in the up-regulation of Gelsolin to prevent cancer cell metastasis (Yuan et al., 2013). Another study reported that stress-induced ATF3–Gelsolin cascade is responsible for spine deficits in the tuberous sclerosis complex (Nie et al., 2015). Knocking down ATF3 expression with shRNA decreased Gelsolin expression and increased dendritic spine density in neuronal models of tuberous sclerosis complex. This relationship between *ATF3-Gelsolin* cascade and spine dynamics under stress conditions inspired us to examine the mRNA expression of *Gelsolin* in *Atf3^-/-^* mice after training. Consistent with previous report (Nie et al., 2015), we found that the expression of *Gelsolin* was downregulated and dendritic spine density was increased in *Atf3^-/-^* mice compared with their wild-type littermates. These results suggest that loss of *ATF3* cause reduced expression of *Gelsolin* and hence a disruption in *Gelsolin* activity, leading to a higher dendritic spine density. To our knowledge, this is the first finding of *ATF3-Gelsolin* relationship *in vivo*.

*Profilin*, another class of actin-binding proteins, promotes actin polymerization at the barbed end by changing the actin nucleotide from adenosine diphosphate to adenosine triphosphate (Ackermann and Matus, 2003; Lamprecht et al., 2006; Hotulainen and Hoogenraad, 2010). *Profilin 1* is expressed at both presynaptic and postsynaptic sites in neurons and plays a role in dendritic spine dynamics (Neuhoff et al., 2005), whereas *Profilin 2* is highly expressed in the brain. Both the actin-capping proteins and the proteins promoting polymerization work to maintain dendritic spines only in the required regions (Hotulainen and Hoogenraad, 2010). Because *Profilin 1* has a function opposing that of *Gelsolin*, we measured its expression and determined that it was down-regulated in the naïve mice group. This finding is not related with enhanced freezing responses nor increase of dendritic spine density. Since *Profilin 1* has no *ATF3* binding site, other factors may be involved in promoting *Profilin* 1 expression and actin polymerization. Further studies are required to identify the factors involved in modifying the expression of *Profilin 1* and *2*, and how they interacting with *ATF3*, maintain actin polymerization dynamics and the balance of fear memory expression.

**FIGURE 6:**
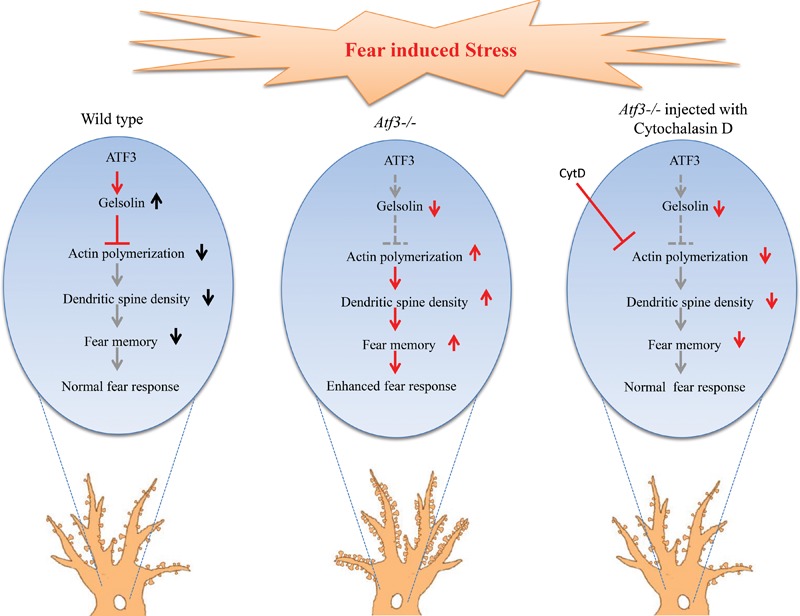
Hypothesized mechanism for *ATF3* modulation of the fear response through actin polymerization and dendritic spine reorganization. Left: *ATF3* modulates normal fear response by increasing the expression of *Gelsolin*, which is an actin polymerization inhibitor. The increase in *Gelsolin* results in decreased actin polymerization and thereby decreases dendritic spine density, resulting in normal fear memory formation. Centre: In the absence of *ATF3*, actin polymerization increases because of *Gelsolin* expression reduction, thereby resulting in increased spine density and higher fear response. Right: Injection of cytochalasin D, an inhibitor of actin polymerization, decreases dendritic spine density and reverses the higher freezing response of the *Atf3^-/-^* to the normal level.

Our results suggests that the *ATF3-Gelsolin* pathway controls the synaptic transmission of fear memory possibly through actin polymerization and maintains fear responses at an optimal level to prevent anxiety disorders (**Figure 6**). Given the role of *ATF3* as a transcription factor, it regulates many genes directly, and affect many others indirectly throughout development. Other pathways regulated by ATF3 are not excluded for their effect on expression of fear memory, but we only checked *ATF3*-*Gelsolin* association in this study. Further delineation of this pathway by identifying other molecules involved in inputting fear cues, activating *ATF3*, and modulating actin polymerization and dendritic spine density will help improve understanding of the molecular mechanism of fear memory formation and the pathology underlying anxiety disorders.

## Author Contributions

IL, C-SP, PS, and H-TH contributed to the hypothesis development, research design, experimental performance, and data analysis. C-SP, SL, and IL wrote the manuscript and prepared the figures for the manuscript. HL provided the knockout mice. Y-LH contributed to the experimental procedures. SP contributed in behavioral experiments and data analysis. All authors read and approved this manuscript.

## Conflict of Interest Statement

The authors declare that the research was conducted in the absence of any commercial or financial relationships that could be construed as a potential conflict of interest.

## Footnotes

1 http://www.ihcworld.com/_protocols/special_stains/golgi_cox.htm
